# High-viscosity injector-based pink-beam serial crystallography of microcrystals at a synchrotron radiation source

**DOI:** 10.1107/S205225251900263X

**Published:** 2019-04-05

**Authors:** Jose M. Martin-Garcia, Lan Zhu, Derek Mendez, Ming-Yue Lee, Eugene Chun, Chufeng Li, Hao Hu, Ganesh Subramanian, David Kissick, Craig Ogata, Robert Henning, Andrii Ishchenko, Zachary Dobson, Shangji Zhang, Uwe Weierstall, John C. H. Spence, Petra Fromme, Nadia A. Zatsepin, Robert F. Fischetti, Vadim Cherezov, Wei Liu

**Affiliations:** a Biodesign Center for Applied Structural Discovery, Biodesign Institute, Arizona State University, 727 East Tyler Street, Tempe, AZ 85287, USA; b School of Molecular Sciences, Arizona State University, 551 East University Drive, Tempe, AZ 85287, USA; c Department of Physics, Arizona State University, 550 East Tyler Drive, Tempe, AZ 85287, USA; d Advanced Photon Source, Argonne National Laboratory, 9700 South Cass Ave, Lemont, IL 90439, USA; e Center for Advanced Radiation Sources, The University of Chicago, Argonne National Laboratory, 9700 South Cass Ave, Lemont, IL 90439, USA; f Department of Chemistry, Bridge Institute, University of Southern California, 1002 Childs Way, Los Angeles, CA 90089, USA

**Keywords:** third-generation synchrotrons, pink-beam serial crystallography, injector-based serial crystallography, structure determination, membrane proteins, protein structures, X-ray crystallography, structural biology

## Abstract

Since the first successful serial crystallography (SX) experiment at a synchrotron radiation source, the popularity of this approach has continued to grow showing that third-generation synchrotrons can be viable alternatives to scarce X-ray free-electron laser sources. Synchrotron radiation flux may be increased ∼100 times by a moderate increase in the bandwidth (‘pink beam’ conditions) at some cost to data analysis complexity. Here, we report the first high-viscosity injector-based pink-beam SX experiments. The structures of proteinase K (PK) and A_2A_ adenosine receptor (A_2A_AR) were determined to resolutions of 1.8 and 4.2 Å using 4 and 24 consecutive 100 ps X-ray pulse exposures, respectively. Strong PK data were processed using existing Laue approaches, while weaker A_2A_AR data required an alternative data-processing strategy. This demonstration of the feasibility presents new opportunities for time-resolved experiments with microcrystals to study structural changes in real time at pink-beam synchrotron beamlines worldwide.

## Abstract 2

This article presents the first injector-based serial crystallography experiments carried out at a pink X-ray beam source, the BioCARS beamline at the Advanced Photon Source. Microcrystals of human A_2A_ adenosine receptor and proteinase K were screened and the structures determined to resolutions of 4.2 and 1.8 Å, respectively.

## 1. Introduction   

In recent years, the field of structural biology has experienced numerous technological breakthroughs that have accelerated protein structure determination. Among these advances are high-brightness photon sources such as X-ray free-electron lasers (XFELs), fast read-out detectors (*e.g.* CSPAD, AGIPD and EIGER) and novel sample-delivery methods (*e.g.* electro­spun liquid microjets, microfluidic devices, GDVN injector and high-viscosity injector) (Ayvazyan *et al.*, 2006 ▸; Feld *et al.*, 2015 ▸; Huang *et al.*, 2015 ▸; Sierra *et al.*, 2012 ▸; Weierstall *et al.*, 2014 ▸, 2012 ▸). Serial femtosecond crystallography (SFX) at XFELs, in which diffraction snapshots are collected from thousands of nano- or microcrystals in random orientations (Chapman *et al.*, 2011 ▸), has solved two major technical challenges of conventional synchrotron single-crystal methods: (1) the need for production of large, well diffracting crystals and (2) inevitable radiation damage associated with X-ray exposures. In addition, SFX enabled time-resolved studies of conformational transitions in proteins with a sub-picosecond resolution allowing insights to be gained into molecular mechanisms of their function (Kupitz *et al.*, 2014 ▸; Mizohata *et al.*, 2018 ▸; Olmos *et al.*, 2018 ▸; Pande *et al.*, 2016 ▸; Tenboer *et al.*, 2014 ▸). However, owing to the scarcity of XFEL facilities, with only five operational worldwide [the Linac Coherent Light Source (LCLS) at the SLAC National Accelerator Laboratory, in Menlo Park, California, USA; the SACLA in Harima, Japan; the European-XFEL in Hamburg, Germany; the Pohang Accelerator Laboratory-XFEL (PAL-XFEL) in Pohang, South Korea; and the Swiss-Free Electron Laser (SwissFEL) at the Paul Scherrer Institute in Villigen, Switzerland] and lack of beam time availability, serial millisecond crystallography (SMX) experiments at microfocus monochromatic beamlines at storage-ring-based synchrotron radiation sources has become a reliable alternative in certain cases. Microfocus beamlines optimized for macromolecular crystallography at third-generation synchrotrons can produce highly brilliant (up to 10^13^ photons s^−1^) and highly focused (as small as 1 µm) X-ray beams. They are also equipped with fast-readout detectors (*e.g.* PILATUS and EIGER), which have enabled a rapid expansion of the SMX approach to many synchrotron facilities, demonstrating its advantages compared with traditional single-crystal crystallography. It has also been shown that room-temperature SMX experiments at synchrotrons can outrun secondary radiation damage (Owen *et al.*, 2012 ▸; Warkentin *et al.*, 2013 ▸, 2017 ▸).

Since the first proof-of-concept SMX study (Gati *et al.*, 2014 ▸), there has been considerable interest in improving this method to increase the spatial and temporal resolution for studying protein structures and dynamics. Typically, synchrotron sources such as undulators and wigglers produce a polychromatic X-ray beam with a broad bandwidth (Δ*E*/*E* ≃ 10^−1^–10^−2^). An X-ray monochromator can select a particular energy and narrow the bandwidth (Δ*E*/*E* ≃ 10^−4^). With monochromatic X-ray beams, only a small fraction of lattice planes diffracts at any given orientation of the crystal. Therefore, the crystal has to be rotated to bring other planes into the diffracting position in order to collect full reflections. A ‘drawback’ of the monochromatic SMX technique is that thousands of diffraction patterns need to be collected from microcrystals for a complete data set because each pattern is essentially a ‘still’ and therefore all observed reflections are only partially recorded. With pink X-ray beams (bandwidth on the order of Δ*E*/*E* ≃ 5 × 10^−2^), a large number of lattice planes diffract simultaneously as the Bragg condition is satisfied for each of the planes by at least one wavelength of the spectrum so that intensities of most reflections are fully recorded (Moffat, 1998 ▸; Ren *et al.*, 1999 ▸). Thus, a much smaller number of diffraction patterns are needed with a pink beam to build a complete data set compared with a monochromatic beam and, therefore, the time and sample volume needed for structure determination are drastically reduced.

Historically, time-resolved macromolecular crystallography at ambient temperature has been conducted using Laue diffraction techniques. The Laue method has been applied to the study of photoreceptor intermediates, ligand photolysis, and allosteric action in heme proteins, enzymatic reactions, ligand–enzyme interactions, viruses and viral drug complexes (Bourgeois & Royant, 2005 ▸; Hajdu & Johnson, 1990 ▸; Neutze & Moffat, 2012 ▸; Šrajer & Schmidt, 2017 ▸; Stoddard, 2001 ▸). Moffat and colleagues have pioneered this field, conducting experiments at CHESS, NSLS, ESRF and the Advanced Photon Source (APS) beamlines (Bourgeois *et al.*, 1996 ▸; Genick *et al.*, 1997 ▸; Ihee *et al.*, 2005 ▸; Knapp *et al.*, 2006 ▸; Moffat *et al.*, 1984 ▸; Ren *et al.*, 2012 ▸; Schmidt *et al.*, 2010 ▸, 2005 ▸). A number of other groups also played a major role in the development of the field and conducted experiments at CHESS (Fülöp *et al.*, 1994 ▸), Daresbury (Hajdu *et al.*, 1987 ▸), NSLS (Bolduc *et al.*, 1995 ▸; Stoddard *et al.*, 1998 ▸), DESY (Schlichting *et al.*, 1990 ▸), the ESRF (Bourgeois *et al.*, 2003 ▸; Schotte *et al.*, 2003 ▸, 2004 ▸; Wöhri *et al.*, 2010 ▸) and the Photon Factory Advanced Ring (Nozawa *et al.*, 2007 ▸). BioCARS at APS specializes in time-resolved pump–probe experiments where short laser pulses are used as the pump and high flux, polychromatic X-ray pulses are used as the probe. The pink beam at the BioCARS beamline, (sector 14) 14-ID-B, with 5.1 × 10^16^ photons s^−1^, offers an average photon flux 100 times higher than the monochromatic beams and is coupled with the 100 ps time-resolution capability, which provides a dependable platform for probing structural intermediates of essential biological macromolecules.

The first proof-of-concept serial crystallography (SX) experiment using a pink X-ray beam at BioCARS was recently reported by Meents *et al.* (2017 ▸). They determined the structures of proteinase K and phycocyanin using 10–20 µm and 30–40 µm sized crystals, respectively, by fixed-target SX using a pink X-ray beam. Here, we report the first proof-of-concept high-viscosity injector-based pink-beam SX experiments using lipidic cubic phase (LCP) as a crystal-carrier medium. Two target proteins were chosen for this pilot experiment: (1) the human A_2A_ adenosine receptor (A_2A_AR) (Liu *et al.*, 2012 ▸), one of the best-studied G protein-coupled receptors (GPCRs); and (2) proteinase K (PK), a commercially available serine protease used in numerous studies to identify disordered regions. The structures of A_2A_AR and PK were determined to resolutions of 4.2 and 1.8 Å, respectively. Laue data-processing software, *Precognition* (Renz Research Inc.), used at BioCARS was able to index the strong images obtained for PK data. However, it could not index A_2 A_AR data that consisted of weak and sparse Laue diffraction patterns. An alternative hit-finding and indexing strategy was necessary to analyze A_2A_AR data, which was shown to be essential when dealing with weak and sparse Laue diffraction patterns from microcrystals.

## 2. Results   

**Figure 1:**
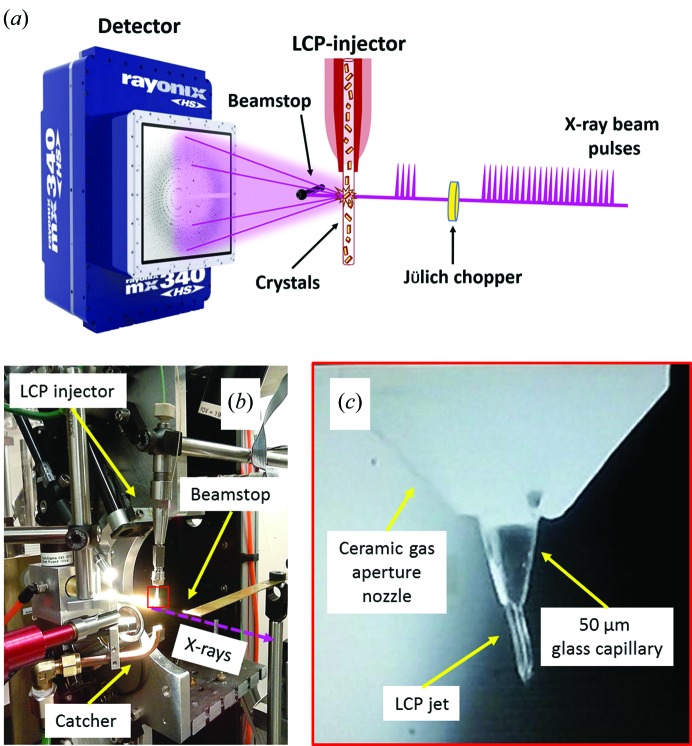
Experimental setup at the BioCARS beamline, 14-ID-B. (*a*) Schematic diagram of the setup. APS was operating in the 24-pulse mode. A Jülich chopper was used to choose the desired number of pulses. A Rayonix detector MX340HS was used to record crystal diffraction. (*b*) LCP injector (Weierstall *et al.*, 2014 ▸) mounted on translation stages. The catcher and beamstop are also shown. (*c*) Closer view of the red boxed area in (*b*) showing the LCP stream extruding out of a glass capillary with a 50 µm internal diameter, which was inserted into a ceramic injection-molded gas aperture nozzle.

To demonstrate the proof-of-concept of the LCP injector-based pink-beam SX at the BioCARS beamline (14-ID-B), we tested two target proteins: A_2A_AR (an integral membrane protein) and PK (a soluble protein). In our study, we used an LCP injector designed by Uwe Weierstall at Arizona State University (Weierstall *et al.*, 2014 ▸) to deliver microcrystals of a size range between 5 µm (A_2A_AR) and 10–15 µm (PK). Microcrystals of PK were first obtained by the batch crystallization method, then pelleted, re-suspended in the crystallization buffer to the desired concentration and finally reconstituted into LCP as previously described (Martin-Garcia *et al.*, 2017 ▸). The resulting PK crystal-laden LCP mesophase was then transferred into an LCP injector. Microcrystals of A_2A_AR were grown in LCP and transferred to an LCP injector directly in the same medium as used for crystallization. Data collection was carried out with APS operating in a 24-bunch mode, in which 24 pulses of 100 ps duration (FWHM) are equally separated by 153 ns. The experimental setup is shown in Fig. 1 ▸. Diffraction data were initially collected using a 25 × 15 µm beam by choosing a single-pulse exposure of 100 ps duration to deliver 7.5 × 10^9^ photons per pulse to a single crystal. However, only relatively low-resolution diffraction patterns were observed, indicating that more photons were needed to obtain a sufficient signal at higher resolution. Subsequently, photon flux was increased by using 4 and 24 consecutive pulses for PK and A_2A_AR, respectively. Complete data sets were recorded and structures of the proteins were determined as we describe below.

### 2.1. Crystal structure of PK in LCP medium with four consecutive X-ray pulses   

PK microcrystals (10–15 µm) embedded into LCP medium were delivered into the pink X-ray beam using a constant flow rate of 29.4 nl min^−1^ with a total data-collection time of 1 h, which resulted in a sample consumption of 2.0 µl. High-quality diffraction data were collected using four consecutive 100 ps pink X-ray beam pulses separated by 153 ns so that each crystal was exposed for a total of 460 ns (actual X-ray exposure time of 400 ps), resulting in a dose per crystal of 30 kGy. A total of 30 000 images were recorded, of which 946 were identified as crystal hits (hit rate 3.1%) and 626 indexed, resulting in a combined indexing and hit rate of 2.1%. The structure of PK was determined to a resolution of 1.8 Å in space group *P*4_3_2_1_2 using only the best 132 patterns out of a total of 626 patterns that were indexed (see Materials and methods[Sec sec5]). The final data-collection and refinement statistics are shown in Table 1 ▸. A Laue diffraction pattern with diffraction up to a resolution of 1.8 Å from a single microcrystal of PK in LCP is shown in Fig. S1A in the Supporting information. The Bragg reflections are well separated and show a subtle elongation in the radial direction indicating a low degree of crystal mosaicity. Phasing was performed by molecular replacement using the room-temperature SMX structure we recently reported from monochromatic data collected at GM/CA (23-ID-D) at APS as a search model [Protein Data Bank (PDB) entry 5uvl; Martin-Garcia *et al.*, 2017 ▸]. The structure of PK with pink-beam SX presented herein was refined to a resolution of 1.8 Å. The final refinement resulted in *R*_work_ and *R*_free_ values of 12.9% and 17.5%, respectively.

**Figure 2:**
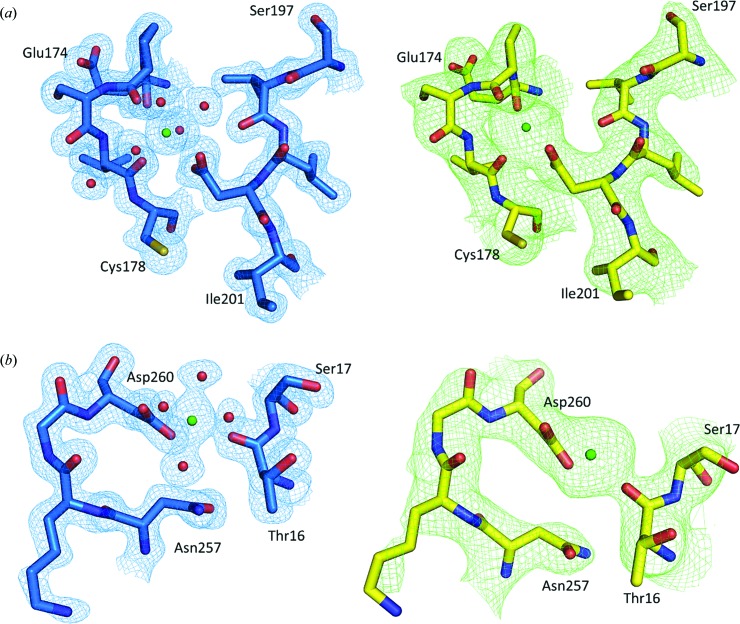
Comparison of the quality of the electron-density maps (2*mF*_o_ − *DF*_c_) of the PK structures determined using a pink beam (left panels) and a monochromatic beam (Martin-Garcia *et al.*, 2017 ▸) (right panels) contoured at 1.5σ. (*a*) Electron-density map around the Ca^2+^ site 1. (*b*) Electron-density map around the Ca^2+^ site 2. Ca^2+^ ions and water molecules are represented as green and red spheres. Residues around Ca^2+^ ions are shown in a stick representation.

**Figure 3:**
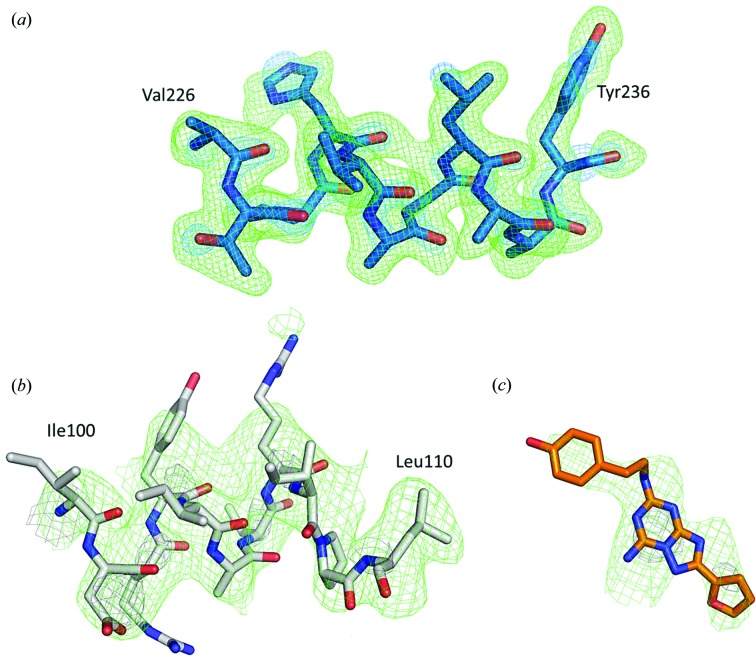
Difference electron-density maps of PK (*a*) and A_2A_AR [(*b*) and (*c*)] structures to illustrate the absence of model bias. Residue fragment Val226–Tyr236 of PK structure (*a*), Ile100–Leu110 of A_2A_AR (*b*) and ligand ZM241385 (*c*) were deleted from models and simulated-annealing OMIT maps 2*mF*_o_ − *DF*_c_ (gray) and *mF*_o_ − *DF*_c_ (green) were calculated. In all cases [(*a*) and (*b*)], OMIT maps 2*mF*_o_ − *DF*_c_ are contoured at 1.5σ and *mF*_o_ − *DF*_c_ are contoured at 2.5σ. Omitted residues are shown for clarity. The positive electron density, where original residues were, is in good agreement with the final structural model of PK and A_2A_AR.

We compared our structure with those obtained by conventional monochromatic diffraction experiments from a single crystal at room temperature (PDB entry 2prk; Betzel *et al.*, 1988 ▸) and from cryo-cooled crystals (PDB entry 5avj; Yazawa *et al.*, 2016 ▸) as well as with the structure recently reported by Meents and co-workers using pink-beam SX on chips (PDB entry 5mjl; Meents *et al.*, 2017 ▸). All r.m.s.d. values are shown in Table S1. Overall, our structure superimposed very well with all PK structures for all C^α^ atoms (r.m.s.d. < 0.18 Å). Superimposition of all atoms (0.35 Å < r.m.s.d. < 0.73 Å) indicates that small differences were, however, observed in the solvent-exposed regions. The average *B* factor is 9.8 Å^2^. The electron-density maps of our pink-beam SX structure show a high level of structural detail. The 136 water molecules identified in our model and the 2*mF*_o_ − *DF*_c_ electron­-density maps around the two Ca^2+^ ions can be used to assess the high quality of our PK structure (Fig. 2 ▸). Furthermore, a comparison of the *mF*_o_ − *DF*_c_ electron-density maps of our structure with those from monochromatic data obtained using SMX (Martin-Garcia *et al.*, 2017 ▸) is shown in Fig. 2 ▸. To ensure that model structural bias is absent in our PK structure, the residue fragment between Ala226 and Tyr236 was deleted from the model, and simulated-annealing OMIT maps were calculated (Terwilliger *et al.*, 2008 ▸). The strong positive peaks visible in place of all omitted residues indicate the absence of model bias in our model (Fig. 3 ▸).

### 2.2. Crystal structure of A_2A_AR in LCP medium with 24 consecutive X-ray pulses   

Because of the small size of the A_2A_AR crystals (5 µm) and the relatively large X-ray beam [25 × 15 µm (*H* × *V*)], only weak diffraction to low resolution was observed using 4 consecutive X-ray pulses indicating that a much higher number of photons were needed to collect a data set from such small crystals. We, therefore, used 24 consecutive pulses so that each A_2A_AR crystal was exposed for a total of 3.53 µs (actual X-ray exposure of 2.4 ns) and received a total of 1.82 × 10^11^ photons and a radiation dose per crystal of 210 kGy. Microcrystals of A_2 A_AR were injected into the X-ray pink-beam path at a constant flow rate of 60 nl min^−1^ and were measured for 7 h with an overall LCP sample consumption of 25.2 µl. A total of 250 000 images were recorded, of which 7363 were identified as hits (3% hit rate) and 771 were successfully indexed (10.5% indexing rate), integrated, scaled and merged in space group *C*222_1_ by *CrystFEL* (version 0.6.3; White *et al.*, 2012 ▸, 2013 ▸, 2016 ▸), resulting in a combined hit and indexing rate of 0.3% (see Material and methods[Sec sec5] and Table 1 ▸ for details).

**Figure 4:**
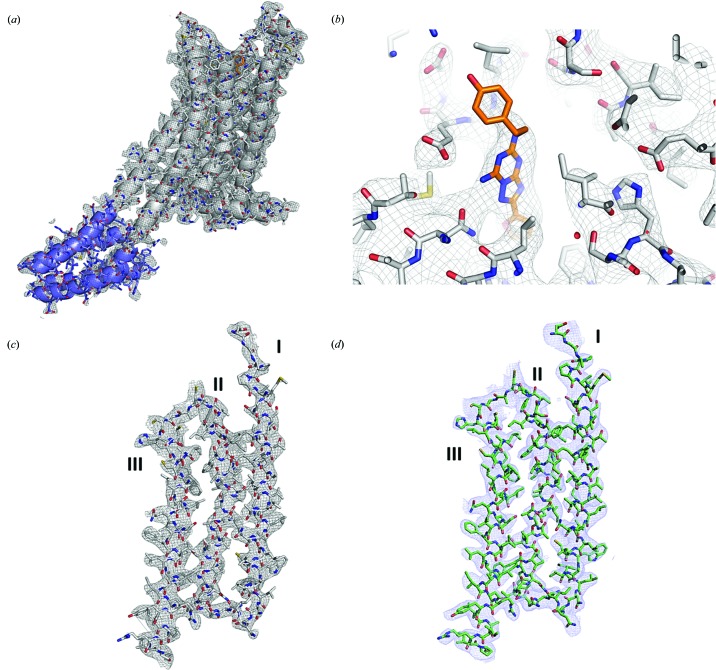
Quality of the electron-density maps of the pink-beam structure of A_2A_AR. (*a*) The A_2A_AR and BRIL fusion proteins are shown as a cartoon and stick representation in white and violet, respectively. (*b*) Difference electron-density maps (2*mF*_o_ − *DF*_c_ at 1.5σ) around the ligand ZM241385 (orange stick representation) and neighboring residues. (*c*) and (*d*) Comparison of the quality of the 2*mF*_o_ − *DF*_c_ electron-density maps (at 1.5σ) around the residues, shown as sticks for helices I, II and III of the pink-beam A_2A_AR structure (*c*) and the monochromatic A_2A_AR structure (*d*) (Martin-Garcia *et al.*, 2017 ▸).

Fig. S1B shows a Laue diffraction pattern from a single A_2A_AR microcrystal. The A_2A_AR structure was solved by molecular replacement using our recently reported SMX structure (PDB entry 5uvi; Martin-Garcia *et al.*, 2017 ▸) without the ligand ZM241385 and lipids. The final structure was refined to a resolution of 4.2 Å with an *R*_work_ and *R*_free_ of 25.0% and 28.8%, respectively. Final data-collection and refinement statistics are shown in Table 1 ▸. Our pink-beam electron-density maps show a relatively high level of structural detail (Fig. 4 ▸). In fact, despite the low resolution of the structure (4.2 Å), the good quality of the electron-density maps allowed us to model the ligand ZM241385 [Fig. 4 ▸(*b*)]. The absence of significant model bias in our A_2A_AR pink-beam structure was demonstrated by deleting the fragment between His100 and Leu110 from the model and the ligand ZM241385, and calculating simulated-annealing OMIT maps. The strong positive peaks visible in place of omitted residues indicate the absence of model bias in our model (Fig. 3 ▸). A comparison of the 2*mF*_o_ − *DF*_c_ electron-density maps of A_2A_AR obtained by pink-beam serial crystallography with those obtained from SMX using monochromatic data (Martin-Garcia *et al.*, 2017 ▸) is also shown in Figs. 4 ▸(*c*) and 4(*d*).

To ensure the validity of our procedure, a comparison of our Laue A_2A_AR crystal structure with high-resolution structures obtained by monochromatic diffraction experiments using either SFX (Batyuk *et al.*, 2016 ▸), cryo-cooled single crystals (Liu *et al.*, 2012 ▸) or SMX (Martin-Garcia *et al.*, 2017 ▸) reveals only small differences between the structures. All structures are very similar to each other, with r.m.s.d. values <0.5 Å for all C^α^ atoms (Table S1). Larger differences were found around the side chains of highly solvent-exposed residues as expected. In addition, structural discrepancies were slightly higher along the backbone in the BRIL fusion protein (Table S1).

### 2.3. Radiation damage   

To evaluate potential site-specific radiation-damage effects on the A_2A_AR structure, we calculated the 2*mF*_o_ − *DF*_c_ and *mF*_o_ − *DF*_c_ electron-density maps and looked at those regions of the proteins that are more susceptible to radiation damage such as disulfide bonds, which were carefully checked for residual electron densities. A_2A_AR has four disulfide bonds (Cys71–Cys159, Cys74–Cys146, Cys77–Cys166, and Cys259–Cys262) in its structure. No significant residual electron densities were found around these disulfide bonds. To further characterize the potential effects of site-specific radiation damage, we calculated the structure-factor amplitude Fourier difference (*F*_o_ − *F*_o_) maps comparing the data sets obtained in this study by serial pink X-ray beam crystallography with the high-resolution structure collected at an XFEL (PDB entry 5k2c; Batyuk *et al.*, 2016 ▸). As shown in Fig. S2, no significant electron-density peaks around any of the disulfide bonds were found at a contour level of 3σ, which indicates that the site-specific radiation damage is negligible or not visible at this resolution and that the disulfide bonds are not completely broken. A similar analysis was carried out with our PK structure. The *F*_o_ − *F*_o_ maps comparing our PK pink-beam structure with the structure from a single cryo-cooled crystal (PDB entry: 5avj; Yazawa *et al.*, 2016 ▸) were calculated and no signs of site-specific radiation damage in the electron density around the two disulfide bridges (Cys34–Cys123 and Cys178–Cys249) were observed (Fig. S3), as expected from the low dose used (30 kGy).

### 2.4. Comparison of A_2A_AR and PK structures from mono- and polychromatic diffraction methods   

We compared the structures of A_2A_AR and PK determined here using pink-beam SX with those we recently obtained using a monochromatic beam at GM/CA (Martin-Garcia *et al.*, 2017 ▸). All data-collection statistics are shown in Table S2. To determine the structure of A_2A_AR to 3.2 Å using a monochromatic beam required a total of 5287 indexed patterns and nearly 14 h of continuous data collection (503 006 snapshots) in which 52.3 µl of sample was consumed at an average flow rate of 56 nl min^−1^ (Martin-Garcia *et al.*, 2017 ▸). The weak Laue diffraction data were difficult to index resulting in a lower indexing rate compared with the monochromatic experiments at GM/CA and thus 250 000 snapshots were recorded at BioCARS to facilitate structure determination (Table S2). In contrast to the experiment at GM/CA, only 7 h and half of the sample volume (25.2 µl) at a similar average flow rate (60 nl min^−1^) were needed to collect a full data set. A comparison of the quality of the electron-density maps for A_2A_AR with a pink beam and a monochromatic beam is shown in Fig. 4 ▸. In the case of PK, eight times fewer indexed patterns [only 132 strong patterns with at least 50 peaks above a signal-to-noise ratio (SNR) of 3σ] were required to determine the high-resolution structure by pink-beam SX compared with using the monochromatic beam at GM/CA (817 indexed patterns) from a sample of microcrystals of similar size and concentration (Table S2).

As pointed out by Meents and co-workers (Meents *et al.*, 2017 ▸; Ren *et al.*, 1999 ▸), the completeness of Laue data sets is typically lower than that of a monochromatic beam. Meents *et al.* attributed this lower completeness to the more challenging data processing, such as resolving spatial overlap of diffraction spots. Difficulty in achieving very high completeness also results from Laue geometry (Ren *et al.*, 1999 ▸). A more conservative SNR cut-off criterion of 3σ is also typically applied to merged Laue data to exclude poorly measured reflections resulting from wavelengths of very low intensity in the incident X-ray spectrum. This also results in lower completeness particularly at higher resolution (Meents *et al.*, 2017 ▸). To assess if the 3σ cut-off applied to our PK pink-beam data was adequate, we also merged data at lower cut-offs of 1.0σ, 1.5σ, and 2.0σ. These trial data sets were processed the same way as the 3σ cut-off data set. Data-collection and refinement statistics for these three data sets are shown in Table 2 ▸. The 2σ or 3σ cut-off as typical cut-offs for Laue data mainly comes from empirical experience in dealing with small signals in time-resolved difference maps. At low sigma cut-offs (resulting in higher *R*_merge_ values), difference maps are typically noisier and difference signal is weaker (as compared with noise). Difference maps therefore become more difficult to interpret and, sometimes, weak signal cannot even be observed. In addition to higher sigma cut-offs in time-resolved experiments, weighting down of poorly measured structure factors is also typically applied, which in a way is reducing completeness further. For an illustration of how weighting affects the time-resolved difference-map signal see Šrajer *et al.* (2001 ▸). Thus, 3σ cut-off was used in our data processing and resulted in improved data quality as judged by the higher refinement *R*_work_ values as well as the higher *R*_work_/*R*_free_ gaps (∼7%).

## 3. Discussion   

In this study, we demonstrated the feasibility of the room-temperature LCP injector-based pink-beam SX at the BioCARS beamline at APS, which can be translated to any other synchrotron sources equipped with pink X-ray beams. We used an LCP injector (Weierstall *et al.*, 2014 ▸) to deliver microcrystals into the 25 × 15 µm (*H* × *V*) pink X-ray beam path. Diffraction data were collected when APS was operating in a 24-bunch mode. We performed experiments using 4 and 24 consecutive pulses. To validate the proof-of-principle of the pink-beam SX approach, we determined the structures of the human adenosine receptor A_2A_AR and the reference protein PK. Despite the much lower resolution of the pink-beam A_2A_AR structure in the present study compared with that obtained at XFELs (Batyuk *et al.*, 2016 ▸), the resulting electron-density maps are of relatively good quality and comparable with those achieved using serial micro-crystallography with a monochromatic beam at a synchrotron source (Martin-Garcia *et al.*, 2017 ▸). In our analysis we find no evidence for site-specific radiation damage and the observed electron densities are not significantly biased despite the low resolution obtained for the A_2A_AR structure.

The most widely used Laue data-processing software, *Precognition* (Renz Research Inc.), was able to index strong images from the PK data set but it could not index weak, streaky and sparse Laue diffraction patterns like those observed for A_2A_AR crystals (Fig. S1). To this end, we adapted the standard pipeline developed for processing monochromatic serial crystallography data using *CrystFEL* with *MOSFLM* (White *et al.*, 2012 ▸, 2013 ▸, 2016 ▸). Our justification in using this pipeline is that in streaky, sparse, pink-beam patterns from small crystals, most measured Bragg peaks are sampled by the brightest part of the spectrum, *i.e.* the central-wavelength photons in the pink-beam distribution, which is critical for monochromatic auto-indexing algorithms. Another reason to use *CrystFEL* with *MOSFLM* for indexing and integration is to evaluate how well the current technology can be adapted to a difficult-to-process Laue data set and to determine where the software limitations lie so we can further develop a customized solution that can be of utility for future SX Laue diffraction experiments. We fully acknowledge that this is not a perfect process – *CrystFEL* with *MOSFLM* was developed for monochromatic beam serial diffraction experiments. Therefore, inherent limitations such as improper peak finding and peak over-prediction are present when grafting the approach to processing Laue diffraction data. These include the absence of typical Laue wavelength scaling prior to intensity merging (see Section 5.3 for a brief discussion). We also only used one wavelength as an input parameter despite pink-beam Laue diffraction experiments being conducted with photons of multiple wavelengths. However, even within such limitations we see acceptable statistics in our A_2A_AR model as presented herein. Our results confirm the reasonable quality of the structure as well as the absence of visible radiation damage. Future developments in data processing of Laue diffraction from crystals measured in a serial fashion will aim to introduce a Laue prediction model, where predicted reflections per pattern are composites of predicted reflections for each wavelength in the Laue spectrum. Such a prediction algorithm automatically assigns a wavelength to each measured Bragg peak, which can then be applied to a wavelength scaled intensity merging algorithm. An illustration of the overall indexing quality of A_2A_AR data is shown in Fig. S5 in which found spots and predicted reflections for each of the ∼770 hits that went into the merge are shown.

In a recent study carried out at a synchrotron (GM/CA beamline at APS) using a monochromatic beam, we reported the high-quality structures of A_2A_AR and PK (Martin-Garcia *et al.*, 2017 ▸) from crystals delivered into the X-ray beam in a serial mode using an LCP injector (Weierstall *et al.*, 2014 ▸). The number of indexed patterns needed for a complete data set was about several thousands, which is comparable with that required in SFX experiments using SASE X-ray beams at XFELs (Batyuk *et al.*, 2016 ▸). In the pink-beam study reported here we have been able to determine the structures of A_2A_AR and PK from microcrystals of similar sizes and concentration using a much smaller number of indexed patterns (a few hundreds). Two benefits of the increased bandwidth of pink beam over monochromatic beam are: (1) reflections falling within the limiting Ewald spheres are fully recorded and (2) more reflections fall within the Ewald sphere (Moffat *et al.* (1984 ▸). However, the SNR of a given reflection decreases if the increasing bandwidth leads to increased intensity because the background scattering is proportional to the incident intensity. The ideal bandwidth to fully record reflections with maximum SNR is probably slightly larger than that required to match the crystal mosaicity. The significant reduction in the number of indexed patterns required to determine the structure was possible because of the significantly wider bandwidth of the pink X-ray beam used at the BioCARS beamline (Δ*E*/*E* = 5 × 10^−2^) which primarily results in reflections being fully recorded.

Primary radiation damage occurs on time scales of 100 fs. Thus, the 100 ps pulse duration at the APS is not fast enough to outrun primary radiation damage as can be done at XFELs. Secondary radiation damage from free radicals diffusing in the crystal and surrounding medium is thought to occur on time scales between microseconds and nanoseconds. The 100 ps pulse is sufficiently short to outrun secondary damage in a single-shot experiment. In these experiments, in order to increase the intensity per exposure the intensity from several (4–24) bunches were accumulated on a single detector frame. The longer the exposure the more likely secondary radiation damage is to be observed. However, for these experiments the total dose and exposure duration were sufficiently low that site-specific radiation damage was not observed. Our single-shot data-collection protocol does not allow us to assess the effects of global damage.

The first pink-beam SX experiment was performed using crystals delivered by a fixed-target method (Meents *et al.*, 2017 ▸). Data were collected with a single 100 ps X-ray pulse exposure per crystal in the APS hybrid mode. Each pulse delivered about 3 × 10^10^ photons to the sample. High-resolution structures of PC and PK were determined from microcrystals between 20 and 40 µm in size, which is similar to or larger than the beam size [20 × 20 µm (*H* × *V*), FWHM] used in their study. Here, we not only explored the minimum crystal size required to collect a complete data set at BioCARS, but also used an LCP injector with the pink beam, confirming that despite the higher background caused by X-ray scattering from the LCP crystal carrier, data of very good quality can be obtained. To this end, different scenarios of data collection by increasing the number of pulses from 1 to 24 were explored using A_2A_AR as a pilot protein with a crystal size as small as 5 µm. We observed very low resolution (∼10 Å) and weak diffraction from A_2A_AR microcrystals using one pulse (7.5 × 10^9^ photons) corresponding to an exposure time of 100 ps. These crystals were observed to diffract to ∼5–7 Å when the number of pulses was increased to four. However, the number of pulses had to be increased to 24 to be able to collect a 4.2 Å resolution data set from such small and weakly diffracting crystals.

It is well known that radiation damage can represent a serious problem when collecting data on small crystals when using synchrotron radiation sources. However, use of the SX technique, in which crystals are replenished sufficiently quickly using new sample-delivery methods (such as the high-viscosity injector), has demonstrated that the effects of radiation damage can be reduced significantly even when measuring microcrystals at room temperature with a monochromatic beam (Beyerlein *et al.*, 2017 ▸; Botha *et al.*, 2015 ▸; Gati *et al.*, 2014 ▸; Hasegawa *et al.*, 2017 ▸; Heymann *et al.*, 2014 ▸; Huang *et al.*, 2016 ▸, 2015 ▸; Martin-Garcia *et al.*, 2017 ▸; Meents *et al.*, 2017 ▸; Murray *et al.*, 2015 ▸; Nogly *et al.*, 2015 ▸; Stellato *et al.*, 2014 ▸; Weinert *et al.*, 2017 ▸; Zander *et al.*, 2015 ▸). In a recent publication on radiation damage (Coquelle *et al.*, 2015 ▸), the disulfide bridges of lysozyme were observed to be damaged by radiation when data sets were collected using a raster-scanning method and much higher doses (3.2 and 29.1 MGy) compared with the theoretical safe dose (0.3 MGy; Nave & Garman, 2005 ▸). In our study, the structures of PK and A_2A_AR were determined using 4 and 24 consecutive pulses, respectively, in which the number of photons delivered was 3 × 10^10^ for the 4-pulse structure, and 1.82 × 10^11^ for the 24-pulse structure. The absence of visible radiation damage in our structures is consistent with the average absorbed radiation dose being significantly lower (Table 1 ▸) than the theoretical safe dose (Nave & Garman, 2005 ▸). Thus, we have demonstrated that the pink X-ray beam SX method described here enables data collection and structure determination from microcrystals at room temperature with non-observable site-specific radiation damage.

Observing biological macromolecules in action at atomic resolution has been the holy grail for structural biologists since the first high-resolution structure of a protein was solved (Blake *et al.*, 1965 ▸). The major challenge in the field of time-resolved X-ray crystallography has been the study of irreversible reactions, which can be initiated, for instance, by a flash of light or by diffusion of a substrate into the crystal lattice of a macromolecule. With the advent of XFELs this is now feasible (Martin-Garcia *et al.*, 2016 ▸). It has recently been demonstrated that the binding of an antibiotic to an enzyme can be studied by collecting data at room temperature using time-resolved SFX at LCLS (Kupitz *et al.*, 2017 ▸; Olmos *et al.*, 2018 ▸). Such studies have remained largely elusive at synchrotron sources because of X-ray radiation damage, the need for growing large single crystals, challenges with crystal replenishment and the difficulty in initiating reactions uniformly in macroscopic crystals. It has been estimated that diffusion of a substrate into microcrystals would take ∼1 ms for 5 µm crystals and 15 ms for 15 µm crystals (Schmidt, 2013 ▸). Thus, crystal sizes used in the study presented here (10–15 µm for PK and ∼5 µm for A_2A_AR) are suitable candidates for time-resolved studies of ligand binding and enzyme catalysis with microsecond resolution at synchrotrons using the pink-beam SX approach. Our results also open new opportunities in the field of GPCRs and other membrane proteins for time-resolved studies of their intermediate states and functional mechanisms at a microsecond and nanosecond resolution. In addition to this, our experimental settings can be extended to other microfocus beamlines at synchrotron ring sources with pink X-ray beams.

## 4. Conclusions and outlook   

Our results demonstrate for the first time the feasibility of using a polychromatic or pink X-ray beam to collect single snapshots from randomly oriented micrometre-sized crystals delivered by a viscous media injector. In similar or equal conditions of crystal quality, size and concentration, by using a broader energy bandwidth of 5% at BioCARS, a much smaller number of diffraction patterns were required to assemble a complete data set compared with monochromatic radiation, which dramatically reduces the amount of sample required for structure determination. However, the 5% bandwidth is probably too wide. The ideal bandwidth is probably slightly wider than that required to record full reflections of optimal SNR without rotating the crystal in the X-ray beam.

Although the processing of pseudo-Laue data using *MOSFLM* and *CrystFEL* has limitations, we were, nevertheless, able to build a model of A_2A_AR at 4.2 Å. This work therefore serves as a foundation for the development of software tools specifically for processing weak pseudo-Laue diffraction data, which could not be processed by *Precognition*.

The upcoming APS upgrade (and upgrades of other third-generation synchrotrons), which will bring a smaller and brighter micro-focused beam (0.5 µm circular beam at monochromatic beamlines such as GM/CA, and <10 µm to a few micrometres at BioCARS) with up to two orders of magnitude higher average flux density along with new developments in beamline optics and the acquisition of faster frame-readout detectors, should enable X-ray structure determination and time-resolved experiments at room temperature by reducing the exposure times. Furthermore, the new upgrades will improve the SNR, which has always been an obstacle in Laue crystallography, reducing the achievable resolution so that serial crystallography experiments from microcrystals as small as 1 µm should also be possible.

Finally, the pink-beam mode would offer a clear opportunity to expand this methodology to XFELs (Dejoie *et al.*, 2013 ▸). The combination of the extraordinary properties of XFELs, which offer exceptionally brilliant, microfocused X-ray pulses, a few femtoseconds in duration, with a high repetition rate and full spatial coherence, along with a broader energy bandwidth, could have an enormous impact in the field of serial femtosecond crystallography especially in time-resolved studies (also referred to as ‘molecular movies’).

## 5. Materials and methods   

### 5.1. Sample preparation   

Microcrystals of A_2A_AR and PK were prepared as previously described (Martin-Garcia *et al.*, 2017 ▸). Crystals of A_2A_AR were obtained in the home laboratory and shipped inside syringes to APS using temperature-controlled containers. Crystallization of PK was carried out onsite at the experimental laboratory of the BioCARS beamline 14-ID-B. Crystal size varied between 5 × 5 × 2 µm for A_2A_AR and 15 × 10 × 5 µm for PK (Table 1 ▸). For PK the crystal density was adjusted before mixing them with LCP so that mainly single-crystal hits were observed. The crystal mixtures were loaded directly from the Hamilton syringe into an LCP injector sample reservoir.

### 5.2. Experimental set-up and serial data collection at the BioCARS 14-ID-B beamline   

Serial data collection was performed at the BioCARS beamline (14-ID-B) at the APS at Argonne National Laboratory (Chicago, Illinois). A detailed description of the BioCARS beamline source can be found in Graber *et al.* (2011 ▸). The source consists of two in-line undulators with periods of 23 and 27 mm that together provide high-flux pink-beam capability at 12 keV as well as first-harmonic coverage from 6.8 to 19 keV. The isolation of single X-ray pulses is accomplished by using a combination of two high-speed X-ray choppers and a millisecond shutter. The choppers rotate continuously and modulate (or chop) the beam in time while the shutter is triggered to open on demand and transmit a single X-ray burst (one bunch or multiple bunches) to the sample. Each high-speed chopper is precisely synchronized with the storage-ring master radio-frequency clock at 351.93 MHz being able to isolate single X-ray pulses at 1 kHz in both hybrid and 24-bunch modes of the APS storage ring. In hybrid mode, each isolated X-ray pulse delivers up to ∼3 × 10^10^ photons to the sample.

Microcrystals in a carrier LCP medium were injected into the intersection with the X-ray pink beam by using the viscous LCP injector (Weierstall *et al.*, 2014 ▸) with a 50 µm inner diameter fused silica capillary (‘nozzle’). This nozzle size was chosen to avoid shearing and breaking up of the crystals during injection, nozzle clogging, as well as to minimize the background scattering from crystal carrier streams. For all our experiments, a 20 µl reservoir was used. The experimental setup during our experiment at BioCARS beamline 14-ID-B is represented in Fig. 1 ▸. The LCP injector was mounted similarly to that used in our previous monochromatic experiment at GM/CA (Martin-Garcia *et al.*, 2017 ▸).

During our experiment, the APS storage ring was operating in the standard 24-bunch mode in which 24 pulses circulate with 4.25 mA per pulse and 153 ns separation between pulses. Each pulse has a duration of 100 ps (FWHM) and delivers about 7.5 × 10^9^ photons to the sample at a peak energy of 12 keV. The number of pulses to be used can be selected and isolated on demand from just one single pulse up to 24 consecutive pulses. The number of pulses in our measurements were from 1 to 24 consecutive pulses so that the number of photons delivered to the sample varied from 7.5 × 10^9^ to 1.82 × 10^11^, and the exposure varied from 100 ps to 3.53 µs. We used the minimum X-ray beam spot size currently achievable at BioCARS 25 × 15 µm (*H* × *V*) (FWHM). Two high-resolution microscope CCD cameras were used to allow for the jet centering. Diffracted X-rays were recorded on a RAYONIX MX340HS CCD detector in the standard 2 × 2 binning mode (acquisition rate of 10 Hz).

The average radiation dose (per crystal) was calculated using the *RADDOSE*-3*D* server (Zeldin, Brockhauser *et al.*, 2013 ▸; Zeldin, Gerstel & Garman *et al.*, 2013 ▸), assuming crystals of dimensions 5 × 5 × 2 µm for A_2A_AR and 15 × 10 × 5 µm for PK, a 25 × 15 µm (*H* × *V*) (FWHM) beam size, a photon flux of 5.2 × 10^16^ photons s^−1^, an energy of 12 keV, and exposure times of 460 ns for PK and 3.53 µs for A_2A_AR. The results of this analysis are summarized in Table 1 ▸.

### 5.3. Data reduction and analysis of serial Laue crystallography   

Laue diffraction data for PK were processed using *Precognition*/*Epinorm* software packages (Renz Research Inc.) and the BioCARS python script *pyPrecognition* was used to automate processing of serial data. The script identifies so-called ‘hits’: images that potentially contain diffraction patterns. The criteria used for selecting hits were: more than 50 peaks detected with more than 30 counts above the background. Each hit was indexed separately using known room-temperature cell parameters and space group. Geometry was then refined for each image, including cell parameters. A total of 30 000 frames were recorded, of which 946 were identified as hits, and 715 images were reported by the program as ‘indexed’. However, on inspection, some of these images were mis-indexed or they were multiple-crystal diffraction patterns and were excluded from further analysis so that only 626 images were processed: integrated to 1.8 Å (using the analytical profile-fitting option in *Precognition*), scaled and merged using an *I*/σ(*I*) of 3 as the cut-off (Table 2 ▸). Additionally, a data set composed of only the best 132 images out of the original 626 images (based on a large number of integrated spots, >800) was scaled and merged using an *I*/σ(*I*) of 3 as the cut-off. This data set illustrates that far fewer images than 626 were sufficient to build a high-quality data set. This was the data set selected for further analysis of the structure of PK and is shown in Table 1 ▸. Statistics for merging at *I*/σ(*I*) of 3 for this 132-image data set are also shown in Table 2 ▸, together with merging at lower *I*/σ(*I*) cut-offs for comparison (1.0, 1.5 and 2.0). The completeness as a function of the resolution for the 132-image data set is shown in Fig. S4 at different values of the *I*/σ(*I*) cut-off.

Laue diffraction data of A_2A_AR was processed using the standard analysis toolkit *CrystFEL* (White *et al.*, 2012 ▸, 2013 ▸, 2016 ▸). The data reduction and analysis of the SX data mainly includes hit finding, indexing and intensity integration. Hit finding was conducted as the first step for data reduction to filter out only the actual crystal diffraction images from the blank ones. The hits were then indexed using *indexamajig* from *CrystFEL* (version 0.6.3; White *et al.*, 2012 ▸, 2013 ▸, 2016 ▸) calling auto-indexing module *MOSFLM* (Powell *et al.*, 2013 ▸). Given that the X-ray beam bandwidth is relatively small and the patterns are sparse (about 20 peaks per pattern), the majority of the observed spots were almost certainly sampled by the peak in the wavelength spectrum, hence indexing usually yielded satisfactory lattice constants. Using the geometry diffraction model for monochromatic indexing, the Miller indices were calculated and assigned to the reflections/peaks that were found in the hit-finding stage. A pattern was only used if more than 50% of its spots overlapped with the prediction. Reflections were merged only if they were observed three or more times. Because the patterns were sparse, most peaks were sampled by the central wavelength, hence wavelength scaling prior to reflection merging was bypassed. A detailed description of the reflection-merging process and a subset of indexed patterns showing found and predicted peaks (Figs. S5 and S6) can be found in the Supporting information. Fig. S5 shows, for 64 indexed patterns, the found spots (blue circles) along with the predicted reflections (orange squares), as well as the resolution cut-off (4.2 Å, green circle). Fig. S6 shows a representative from the 771 indexed patterns. More representative indexed patterns can be found in the Supporting information.

### 5.4. Model building and structure refinement   

MTZ files for phasing and refinement were generated by the *CTRUNCATE* program (Padilla & Yeates, 2003 ▸) from the *CCP*4 software package (Winn *et al.*, 2011 ▸). Initial phases were obtained by molecular replacement with *MOLREP* (Vagin & Teplyakov, 1997 ▸) using known structures of the proteins from the PDB. The PDB entries used in our study were 5uvi and 5uvl for A_2A_AR and PK, respectively (Martin-Garcia *et al.*, 2017 ▸). Water molecules and ligands were removed from the reference structures for the phasing step. Structure refinement was carried out through multiple iterations of *REFMAC*5 (Murshudov *et al.*, 2011 ▸) and *phenix.refine* (Winn *et al.*, 2011 ▸; for A_2A_AR) to refine atomic coordinates and isotropic *B* factors. Final refinement of A_2A_AR was also performed with *REFMAC5*. Manual inspection of the structures was carried out using *Coot* (Emsley & Cowtan, 2004 ▸) after each refinement step. The figures were prepared with *PyMOL* (version 1.8, Schrödinger LLC). Data-refinement statistics for all structures solved in this study are summarized in Table 1 ▸. Electron-density and composite OMIT maps were calculated with the *MAPS* tool in the *PHENIX* software suite (Adams *et al.*, 2010 ▸). Validation of all structures was carried out with the validation tools in the *PHENIX* software suite (Adams *et al.*, 2010 ▸).

## Supplementary Material

Supporting information - figures, tables, patterns and merging results. DOI: 10.1107/S205225251900263X/ti5014sup1.pdf

PDB reference: proteinase K, 6mh6

PDB reference: human A_2A_ adenosine receptor, 6mh8

## Figures and Tables

**Table 1:** Data-collection and refinements statistics Values in parentheses are for the highest resolution shell.

	A_2A_AR†	PK‡
Crystal size (µm)	5 × 5 × 2	15 × 10 × 5
Crystal-to-detector distance (mm)	250	250
Duration (h)	7	∼1
Sample flow rate (nl min^−1^)	60	29.4
Exposure time (µs)	3.53	0.46
No. of bunches	24	4
Radiation dose per crystal (kGy)	210	30
Protein/carrier volume (µl)	25.2	2.0
Maximum resolution observed (Å)	3.5	1.8
Resolution (Å)	50.0–4.2 (4.3–4.2)	57.8–1.8 (1.85–1.8)
Space group	*C*222_1_	*P*4_3_2_1_2
*a*, *b*, *c* (Å)	40.0, 179.0, 142.0	68.3, 68.3, 108.3
α, β, γ (°)	90, 90, 90	90, 90, 90
No. of collected images	250000	30000
No. of hits/indexed patterns	7363/771	946/626 (132)§
〈*I*/σ(*I*)〉 (SNR)	4.5 (4.2)	23.3 (13.3)
Multiplicity	11.5 (8.1)	9¶
Completeness (%)	87.8 (78.9)	61.3 (13.7)††
CC* (%)	94.3 (80.0)	N/A‡‡
*R*_split_ (%)	27.0 (26.2)	10.2¶
Total No. of reflections	4021	14910
No. of reflections in refinement	3179	13447
No. of free reflections in refinement	404	1463
*R*_work_/*R*_free_ (%)	25.0/28.8	12.9/17.5
No. of atoms	3003	2176
Protein	2979	2033
Water and others (ligands or ions)	24	143
Average *B* value (Å^2^)	59.3	9.8
R.m.s deviations from ideal values		
Bonds (Å)	0.006	0.02
Angles (°)	1.0	1.8
Ramachandran plot statistics (%)		
Favored	97.4	97.5
Allowed	2.6	2.5
Disallowed	0	0
Rotamer outliers	0	0
PDB entry	6mh8	6mh6

† The structure of A_2A_AR determined from Laue data processed using *CrystFEL*.

‡ The structure of PK determined from Laue data processed using program *Precognition* (Renz Research Inc.)

§ Statistics below are from merging the best 132 indexed images. Images were selected based on the highest number of detected reflections from the total of 626 indexed images.

¶ Only overall multiplicity and *R*_merge_ were reported by *Precognition*. Listed is *R*_merge_(*F* ^2^).

†† Completeness listed is for data merged at 3σ level, standard experience-based cut-off for merging Laue data processed with *Precognition* as a compromise between *R*_merge_ and last shell completeness. In addition, *Precognition* uses a resolution-dependent wavelength bandwidth (Ren *et al.*, 1999 ▸) to prevent overprediction of data at high resolution.

‡‡ Not reported by *Precognition* (Renz Research Inc.).

**Table 2:** Data-collection statistics of PK data to 1.8 Å at different *I*/σ cut-offs used for data merging The structure of PK was determined from Laue data processed using program *Precognition* (Renz Research Inc.). Values in parentheses are for the highest resolution shell.

	1.0σ	1.5σ	2.0σ	3.0σ	3.0σ
No. of collected images	30000	30000	30000	30000	30000
No. of hits/indexed patterns	946/626 (132)†	946/626 (132)†	946/626 (132)†	946/626 (132)†	946/626‡
I/σ (SNR)	13.2 (7.3)	16.4 (8.7)	19.1 (10.2)	23.3 (13.3)	30.4 (12.6)
Multiplicity§	12	10.4	9.8	9	25.8
Completeness (%)	95.3 (71.9)	88.5 (52.1)	77.7 (34.8)	61.3 (13.7)	63.4 (15.6)
*R*_merge_ (*F* ^2^) (%)§	19.3	15.4	13	10.2	12.5
No. of reflections	23224	21477	18860	14910	15446
No. of reflections in refinement	20945	19366	16989	13447	13447
*R*_work_/*R*_free_ (%)[Table-fn tfn11]	22.8/25.7	23.5/25.9	22.1/24.1	17.13/20.41	19.76/22.20
*R*_work_/*R*_free_ (%)[Table-fn tfn12]	17.4/24.2	17.6/24.1	15.0/22.0	12.9/17.5	N/A†

† Statistics below are from merging of best 132 indexed images. Images were selected based on the highest number of detected reflections (>800) from the total of 626 indexed images.

‡ Statistics below are from merging all of the 626 indexed images.

§ Only overall multiplicity and *R*_merge_ are reported by *Precognition*.

¶ Values after initial refinement without solvent molecules.

†† Values of final model after adding all solvent molecules.

‡‡ Structure was determined using 132 indexed paterns.
