# Supplementary material for: High-viscosity injector-based pink-beam serial crystallography of microcrystals at a synchrotron radiation source
Source: IUCrJ. 2019 Apr 5;6(Pt 3):412–25. doi: 10.1107/S205225251900263X (PMC6503920; doi:10.1107/S205225251900263X)
Supplement: Supplementary file 1 [file m-06-00412-sup1.pdf]

IUCrJ

Volume 6 (2019)

Supporting information for article:

High-viscosity injector-based pink-beam serial crystallography of microcrystals at a synchrotron radiation source

Jose M. Martin-Garcia, Lan Zhu, Derek Mendez, Ming-Yue Lee, Eugene Chun, Chufeng Li, Hao Hu, Ganesh Subramanian, David Kissick, Craig Ogata, Robert Henning, Andrii Ishchenko, Zachary Dobson, Shangji Zhang, Uwe Weierstall, John C. H. Spence, Petra Fromme, Nadia A. Zatsepin, Robert F. Fischetti, Vadim Cherezov and Wei Liu

Supporting Information

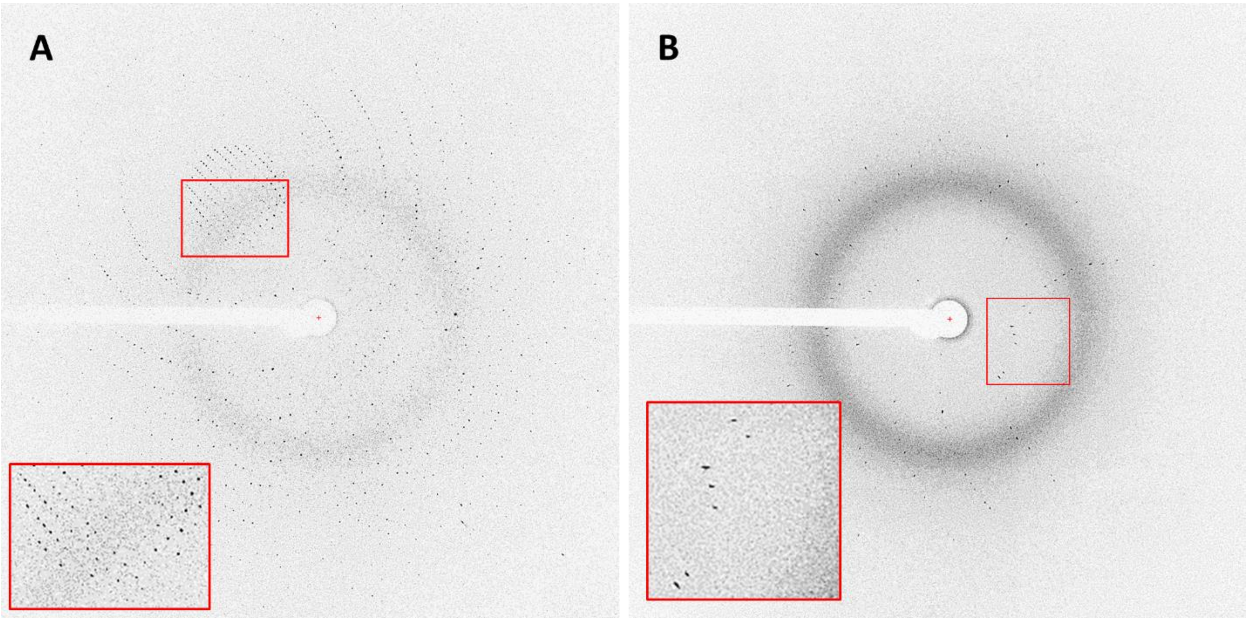

Figure S1. Laue diffraction pattern of a single crystal of PK (A) and A_{2A}AR (B). Crystal sizes were between 10 and 15 µm for PK and about 5 µm for A_{2A}AR. Micro-crystals of both proteins were delivered in LCP as it is shown by the LCP ring at about 4.5 Å on the detector. The insets highlight the shape of the Bragg reflections.

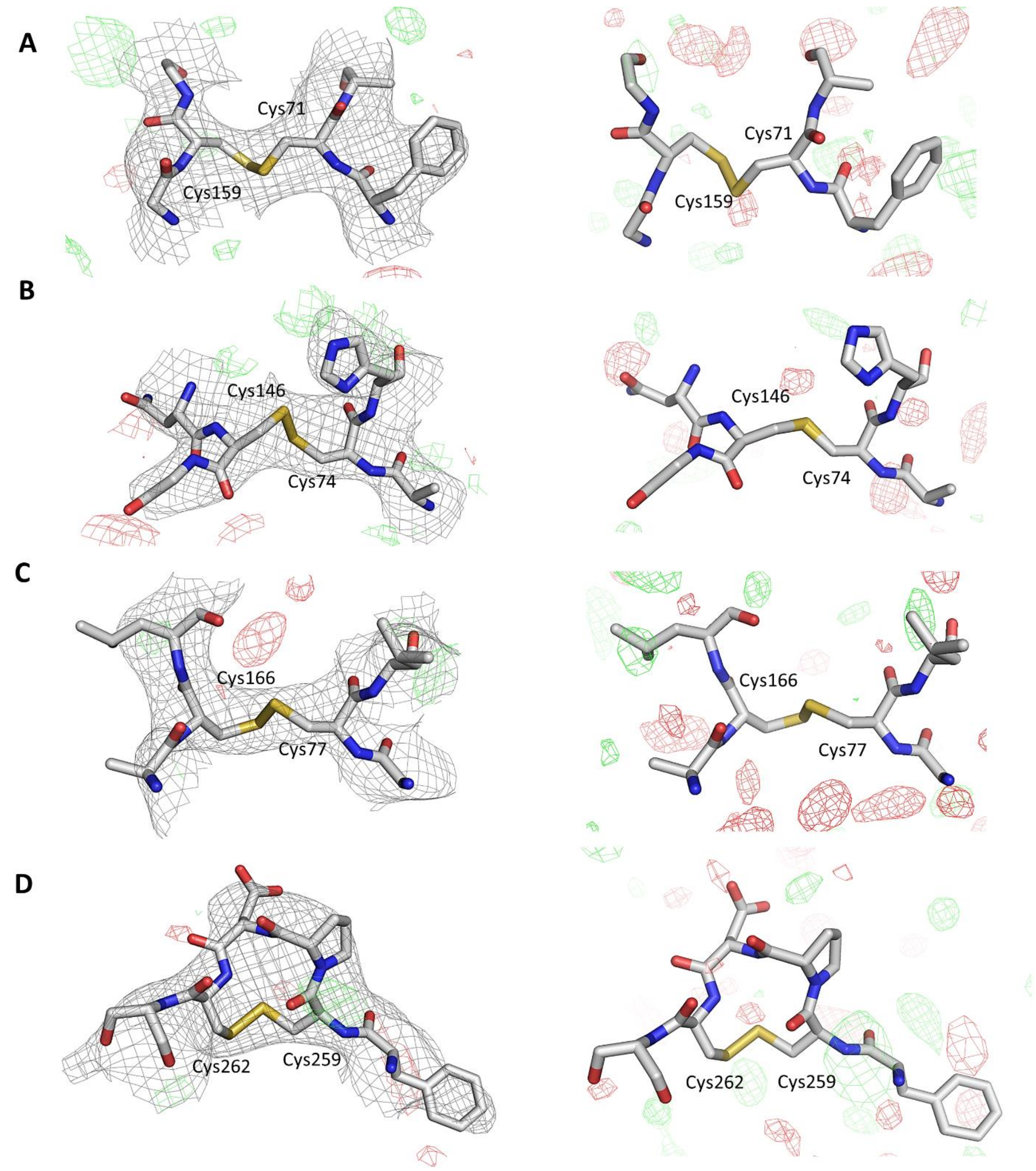

Figure S2. Electron density maps displayed around the four disulfide bridges of A2AAR (Cys71-Cys159 (A), Cys74-Cys146 (B), Cys77-Cys166 (C), Cys259-Cys262 (D)). Cysteines and neighboring residues are shown as stick representation. Left panels: 2mFo-DFc (light grey mesh, contoured at 1.5 σ) and mFo-Fc (red and green meshes, contoured at -/+ 2.5 σ, respectively) maps. Right panels: Structure factor amplitude Fourier difference (Fo-Fo) maps at -/+ 2.5 σ between our data set and the data set collected at LCLS (PDB 5K2C) (Batyuk et al., 2016), with red and green contours indicating negative and positive density, respectively.

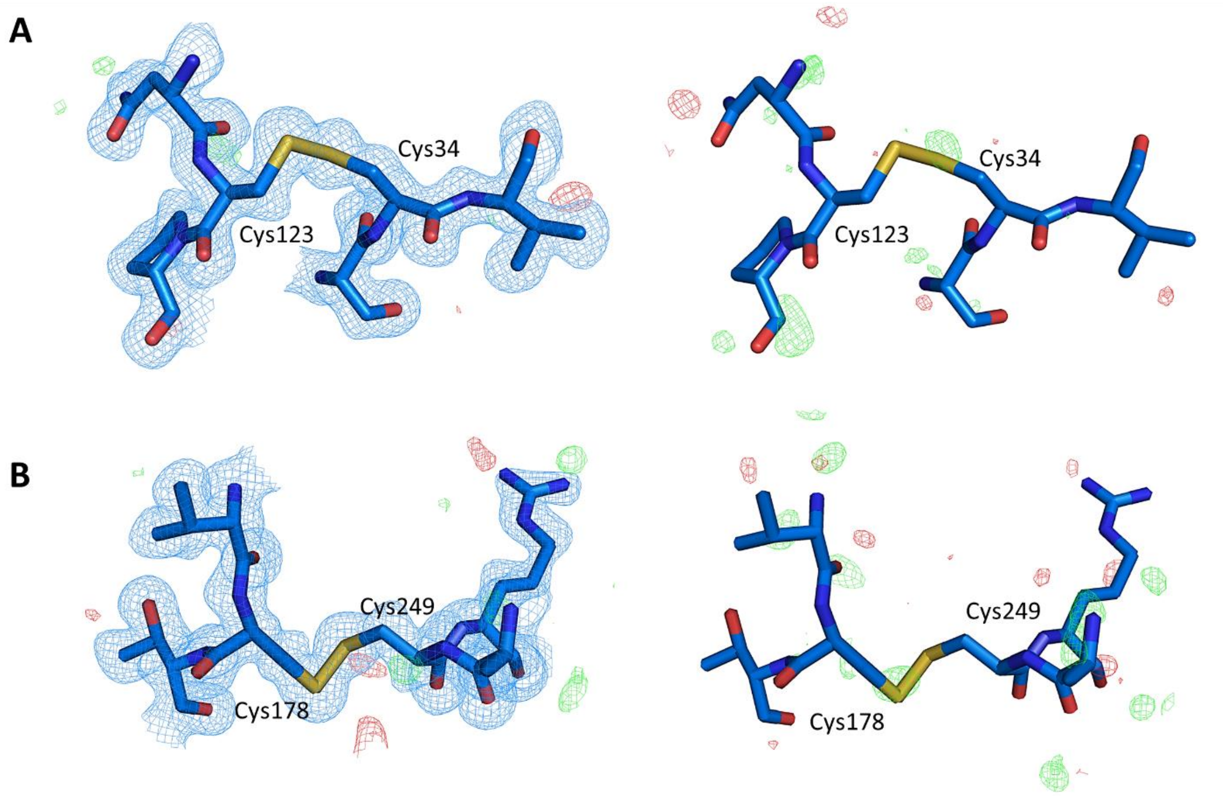

Figure S3. Electron density maps displayed around the two disulfide bridges of PK (Cys34-Cys123 (A), Cys178-Cys249 (B). Cysteines and neighboring residues are shown as stick representation. Left panels: 2mFo-DFc (blue mesh, contour at 1.5 σ) and mFo-Fc (red and green meshes, contoured at -/+ 2.5 σ, respectively) maps. Right panels: Structure factor amplitude Fourier difference (Fo-Fo) maps at 2.5 σ between our data set and the data set collected from cryo-cooled crystals (PDB 5AVJ) (Yazawa, 2016), with red and green contours indicate negative and positive density, respectively.

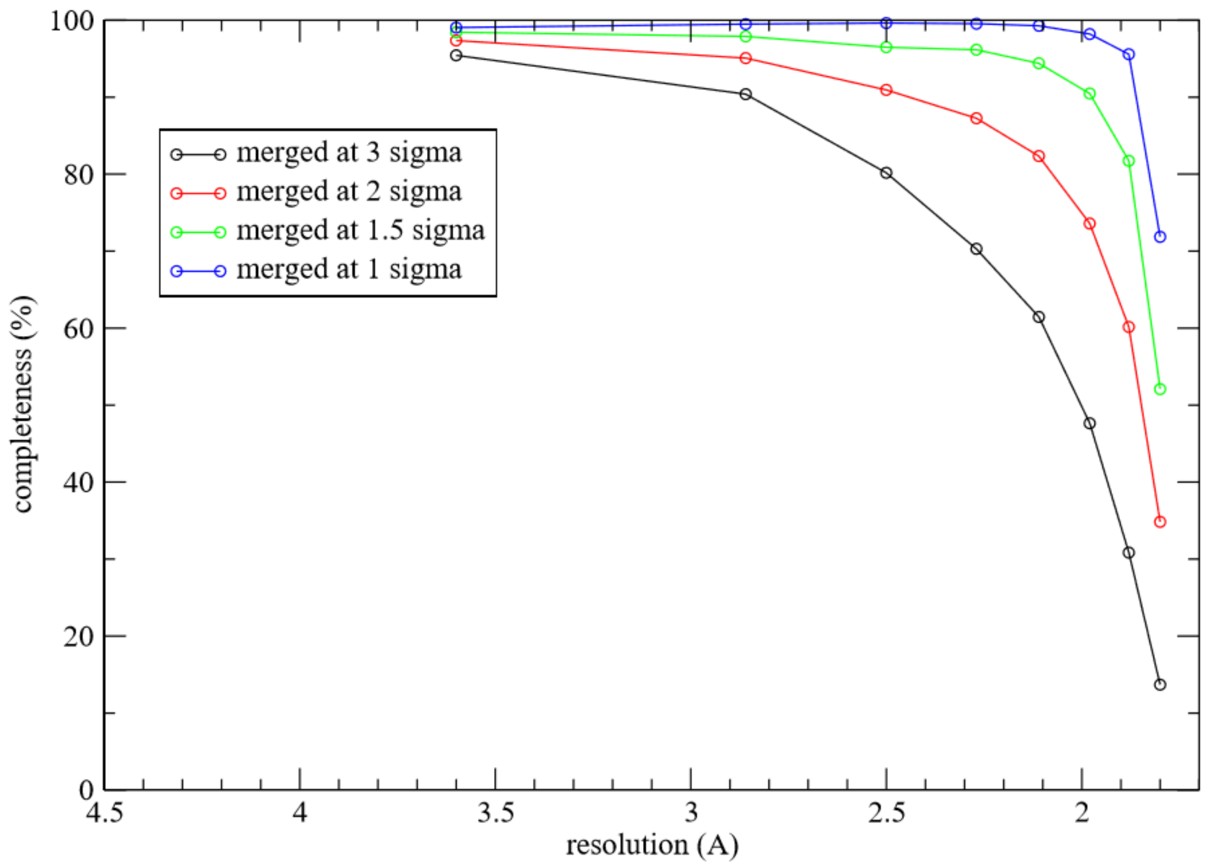

Figure S4. Completeness vs resolution of PK data 132-image data set to 1.8 Å at four I/σ(I) cut off.

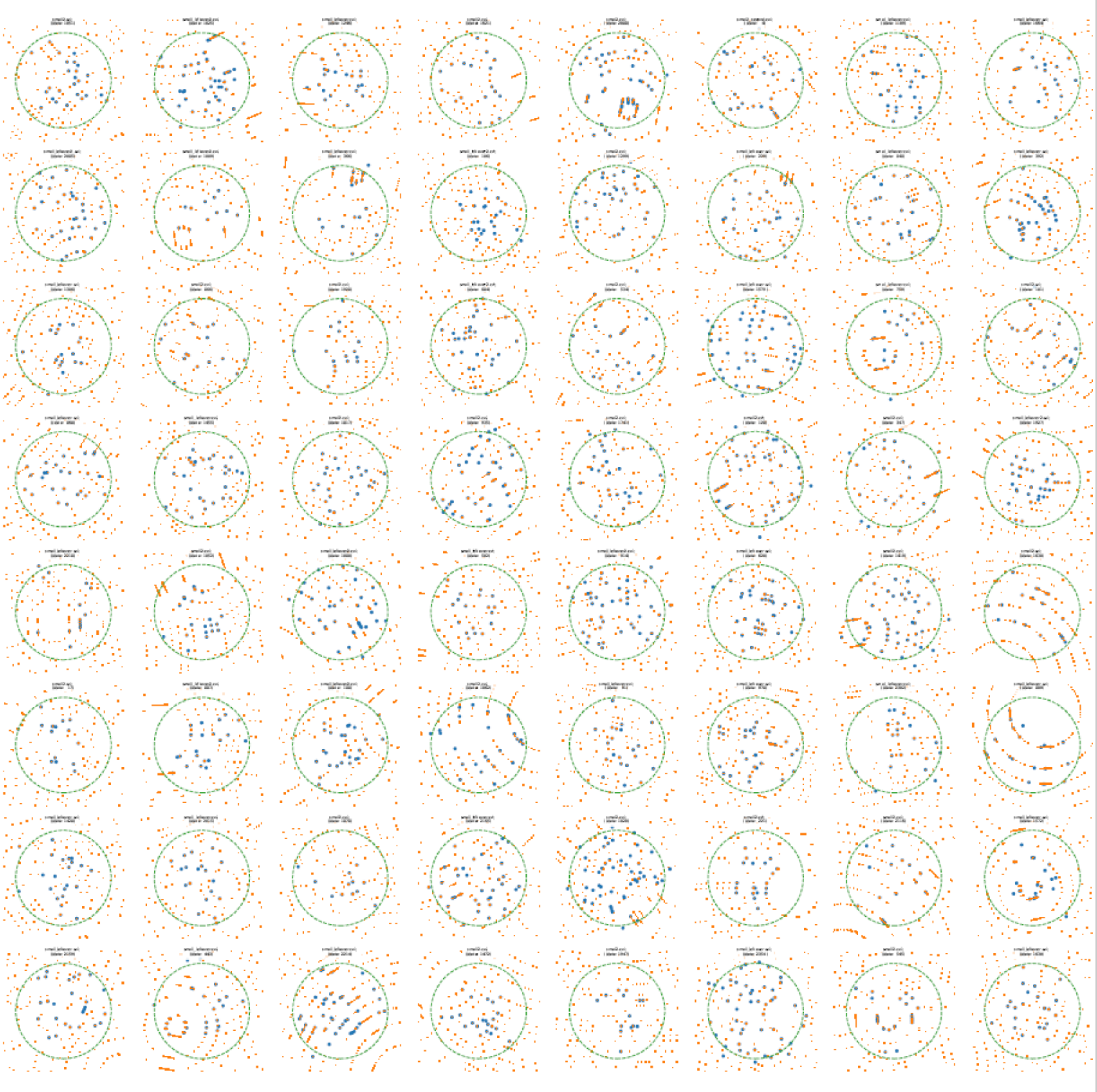

Figure S5. Selected A2aAR indexed patterns. It illustrates an over-all feel for the indexing quality, as it shows, for each and every indexed pattern, the found spots (blue circles) along with the predicted reflections (orange squares), as well as the resolution cutoff (4.2 Angstrom, green circle). The complete list of patterns has been deposited as separate pdf file.

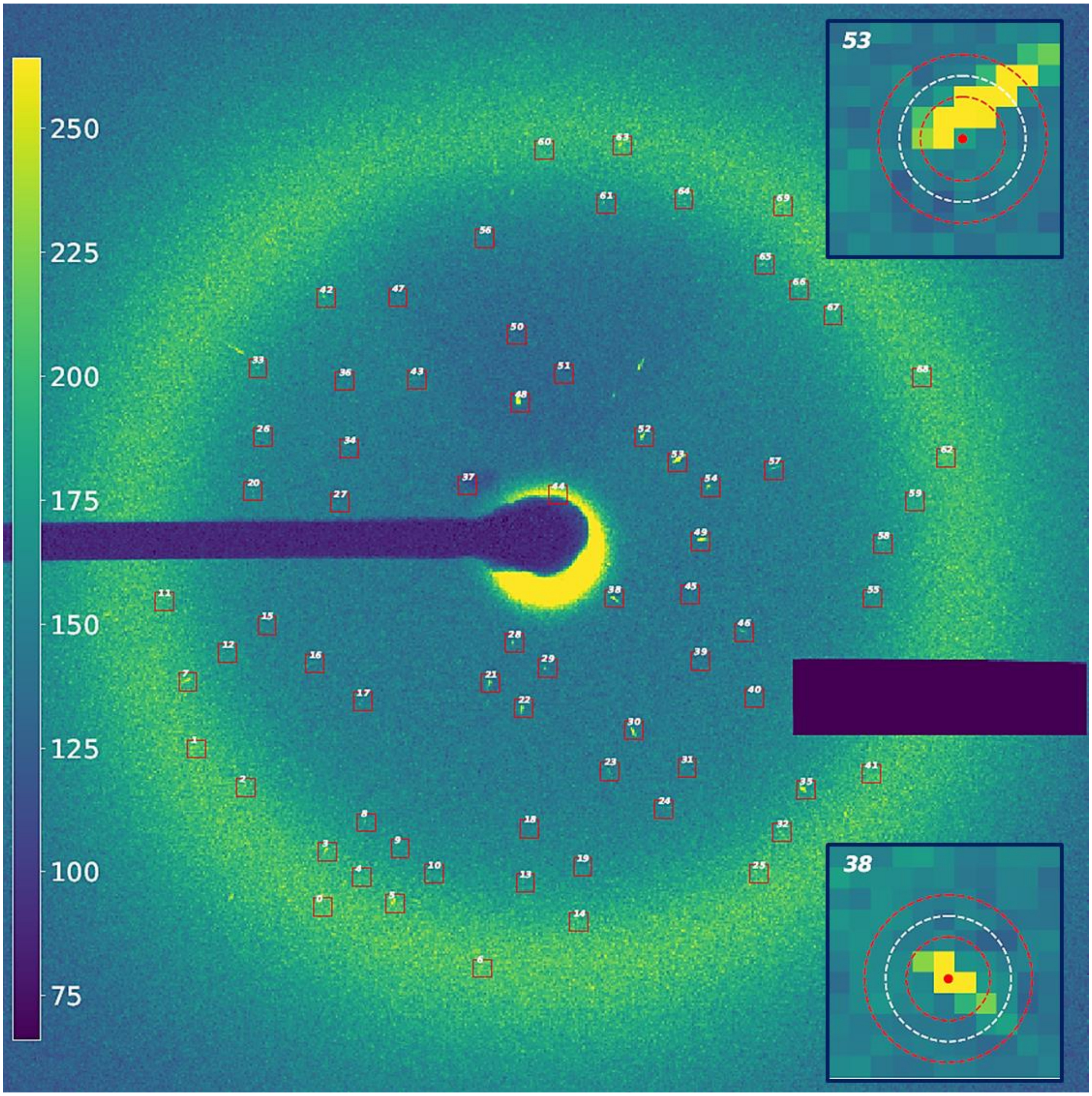

Figure S6. Representative Laue diffraction pattern of $A_{2A}AR$. Predicted peak locations were red boxed. All predicted peaks were also numbered. Inset panels show a zoom in of two random predicted reflections (38 and 53) form the main image, along with the ring radii used in indexing. The inner most ring defines the “signal” region, and the area common to the two outer rings “shell” defines the background region. A few more patterns showing the peak locations and the zoomed in version of each predicted peak along with the ring radii can be in a separate file in supporting information.

Table S1. R.M.S.D. values comparison.

	C^{α} atoms (Å)	All atoms (Å)
PK vs 2PRK	0.115	0.723
PK vs 5AVJ	0.177	0.447
PK vs 5UVJ	0.133	0.409
PK vs 5MJL	0.167	0.349
A_{2A}AR vs 4EIY	0.414	0.752
A_{2A}AR vs 5K2C	0.395	0.617
A_{2A}AR vs 5UVI	0.288	0.460
BRIL vs 4EIY	0.687	1.270
BRIL vs 5K2C	0.556	0.969
BRIL vs 5UVI	0.325	0.432

Table S2. Data collection statistics comparison.

	A_{2A}AR†	PK*	A_{2A}AR_GM/CA	PK_GM/CA
Crystal size (µm)	5 x 5 x 2	15 x 10 x 5	5 x 5 x 2	15 x 10 x 5
Delivery medium	LCP	LCP	LCP	PEO
Duration (h)	7	~1	~14	~3
Sample flow rate (nL/min)	60	29.4	56	79
Exposure time	3.53 µs	0.46 µs	18 ms	15 ms
No. of bunches	24	4	n/a[#]	n/a[#]
Radiation dose per crystal (kGy)	210	30	100	100
Protein/carrier volume (µL)	25.2	2.0	52.3	13.0
Maximum Resolution observed (Å)	3.0	1.8	3.1	2.2
Resolution (Å)	4.2	1.8	3.2	2.65
Space group	$C222_1$	$P4_32_12$	$C222_1$	$P4_32_12$
a, b, c (Å)	40.0, 179.0, 142.0	68.3, 68.3, 108.3	39.4, 179.5, 140.3	68.3, 68.3, 108.2
α, β, γ (°)	90, 90, 90	90, 90, 90	90, 90, 90	90, 90, 90
No. of collected images	250,000	30,000	503,006	97,772
No. of hits/indexed patterns	7,363/771	946/626 (132)**	14,711/5,287	4,497/817

†Structure of A_{2A}AR determined from Laue data processed using CrystFEL[60].
*Structure of PK determined using Precognition (Renz Research Inc.).
**Statistics below are from merging the best 132 indexed images from the total of 626 indexed images. Images were selected based on highest number of detected reflections (>800).
Data collected in a shutterless mode in which continues X-ray beam was hitting the sample.
